# Sequence type and strain-level detection of Klebsiella pneumoniae in culture-enriched bacterial metagenomes: comparative performance of mSWEEP and StrainGE bioinformatic tools

**DOI:** 10.1099/mgen.0.001638

**Published:** 2026-02-11

**Authors:** Dorota Julia Buczek, Wasifa Kabir, Kenneth Lindstedt, Tommi Mäklin, Harry A. Thorpe, Yutaka Suzuki, Jukka Corander, Ørjan Samuelsen, Arnfinn Sundsfjord

**Affiliations:** 1 Department of Medical Biology, Faculty of Health Sciences, UiT the Arctic University of Norway, Tromsø, Norway; 2 ELIXIR Norway, UiT the Arctic University of Norway, Tromsø, Norway; 3 Norwegian Centre for Detection of Antimicrobial Resistance, Department of Microbiology and Infection Control, University Hospital of North Norway, Tromsø, Norway; 4 Department of Biostatistics, University of Oslo, Oslo, Norway; 5 Department of Computational Biology and Medical Sciences, The University of Tokyo, Tokyo, Japan; 6 Helsinki Institute for Information Technology HIIT, Department of Mathematics and Statistics, University of Helsinki, Helsinki, Finland; 7 Parasites and Microbes, Wellcome Sanger Institute, Hinxton, Cambridgeshire, UK

**Keywords:** *Klebsiella pneumoniae*, metagenome, mSWEEP, sequence type, StrainGE

## Abstract

*Klebsiella pneumoniae* is a major cause of human infections and is frequently associated with antimicrobial resistance (AMR). Carriage of *K. pneumoniae* in the gut is a major risk factor for infection and a reservoir for the spread of high-risk clonal lineages and associated AMR determinants. Accurate detection of *K. pneumoniae* at the subspecies level is therefore essential to better understand *K. pneumoniae* gut colonization ecology and clonal dissemination. We analysed two recently developed bioinformatic tools, mSWEEP and StrainGE, for sequence type (ST) detection of *K. pneumoniae* in culture-enriched sweep metagenomes compared to single-colony whole-genome sequencing (WGS). We show that both mSWEEP and StrainGE perform highly accurate ST detection, concordant with culture in 46/49 and 44/49 samples with WGS-detected single STs, respectively, as well as in 2/3 samples with two WGS-detected STs. Within-sample ST diversity was detected in 19 and 15 samples by mSWEEP and StrainGE, respectively, highlighting a major advantage of these tools over conventional single-colony WGS. StrainGE could also reconstruct accurate phylogenetic relationships between strains of the same ST for 2/3 different STs tested. Additionally, assembly of the genomes provides better resolution of ST detection by mSWEEP. Together, our results show that both mSWEEP and StrainGE are accurate tools for the detection and analysis of *K. pneumoniae* STs from mixed bacterial samples.

**Impact Statement**

*Klebsiella pneumoniae* is a major public health threat due to its ability to carry and disseminate antimicrobial resistance (AMR) genes. Gut carriage of *K. pneumoniae* is a major risk factor for infections, making accurate strain-level identification directly from metagenomic samples critical for surveillance and prevention. Our study provides insights into the suitability of two strain-level detection tools from metagenome samples, mSWEEP and StrainGE. By comparing culture-enriched sweep metagenomes with single-colony whole-genome sequencing, we show that both tools can detect strains accurately but differ in computational resource demands and downstream analysis. StrainGE requires less computational resources than mSWEEP, making it a convenient tool to use for rapid identification during outbreak situations. Additionally, while StrainGE supports accurate construction of phylogenetic relationships, it lacks the metagenomic binning included in mSWEEP, limiting further downstream analysis. This highlights the importance of tool selection based on user preference such as research or surveillance.

## Data Summary

All data related to this work are reported in the article. Metagenomic data (raw MGI reads with human residues removed) are publicly available in ENA under BioProject PRJEB87654.

## Introduction

*Klebsiella pneumoniae* (Kpn) is a gram-negative bacterium occupying a wide range of environmental niches. In humans, Kpn acts as a commensal and an opportunistic pathogen colonizing the gastrointestinal tract, causing infections of the urinary tract, respiratory tract and bloodstream in immunosuppressed patients, neonates and elderly [1,6]. Kpn is considered an important reservoir and vector in the dissemination of antimicrobial resistance (AMR), and gut carriage is the most important risk factor for clinical infections [6,8].

Kpn is a part of a wider *K. pneumoniae* species complex (KpSC) including seven phylogroups: *K. pneumoniae sensu stricto* (Kp1), *K. quasipneumoniae* subsp. *quasipneumoniae* (Kp2) and subsp. *similipneumoniae* (Kp4), *Klebsiella variicola* subsp. *variicola* (Kp3) and subsp. *tropica* (Kp5), *K. quasivariicola* (Kp6) and *K. africana* (Kp7) [5,9,12].

Studies of KpSC genomics have shown a highly diverse, but structured population consisting of deep-branching lineages of clonal groups (CGs) [5]. Two subsets of CGs are of particular clinical importance; those associated with the development of multidrug resistance and hypervirulence (HyV). For both subsets, the human gut ecosystem is considered an important reservoir.

However, we have limited knowledge on the diversity and dynamics of Kpn in human gut colonization. Culture-based population studies of healthy adults in Asia have shown a wide range in Kpn gut carriage rates (18–87%) [13,14]. Similar culture-based studies of hospitalized patient in Australia, the UK and the USA have revealed Kpn colonization rates between 11 and 23% [6,15, 16]. A cross-sectional culture-based study of KpSC gut carriage in a Norwegian community-based large human adult general population cohort (*n*=2975; The Tromsø seven study; T7) showed a colonization prevalence of 16.3% and a high Kpn population diversity [17].

Standard culture-based detection methods in the study of Kpn gut carriage and ecology have limitations [3]. The gut bacterial communities contain not only complex mixtures of species but also a mixture of strains within species [18,19]. Interestingly, recent progress in whole metagenomic sequencing (WMS) and bioinformatics has shown promising results in the ability to resolve bacterial microbiota complexity beyond the level of genus and species, supporting sequence type (ST) and strain-level detection [18,19].

The primary aim of this study was to explore and compare two recently developed bioinformatic tools, mSWEEP [18] and Strain Genome Explorer (StrainGE) [19], in their abilities to identify the presence and diversity of Kpn strain STs in culture-enriched sweep mixed bacterial communities. We take advantage of Kpn culture-positive sample material and single-colony whole-genome sequencing (WGS) data from the previous T7 study [17].

## Methods

### Human faecal samples and corresponding whole-genome sequenced Kpn strains

The faecal samples (*n*=52) in this study were selected from KpSC culture-positive specimens within the 2975 KpSC SCAI (Simmons citrate agar with inositol) culture-screened faecal samples obtained during the cross-sectional T7 study [17]. Briefly, faecal samples were plated onto SCAI media (Sigma-Aldrich, Darmstadt, Germany) and incubated for 48 h at 37 °C [20]. Large, yellow, glossy colonies were selected as likely *Klebsiella* spp. and identified to the species level using MALDI-TOF MS (Bruker Daltonics, Bremen, Germany). The first colony identified as KpSC was kept for further analysis, except for three samples in which both *K. pneumoniae* and *K. variicola* isolates were identified in the same sample and kept for further analyses. DNA extraction, WGS and bioinformatic analysis were performed as described [19].

### SCAI-sweep samples for WMS

The selected KpSC culture-positive samples (*n*=52), with corresponding WGS single KpSC isolates from the T7 study, were selected for SCAI-sweep enrichment-based WMS analysis as described [3]. Briefly, 50 µl from thawed ESwab samples was plated on SCAI media (Sigma-Aldrich). Following incubation at 37 °C for 48 h, all growth on plates was scraped, and one loaded 10 µl inoculation loop was used as input for DNA extraction using the Purelink Microbiome DNA purification kit (Thermo Fisher Scientific, Waltham, USA) with minor modifications as described [3]. DNA quality control was performed using NanoDrop 2000 spectrophotometer (Thermo Fisher Scientific) and Qubit 3.0 fluorometer (Thermo Fisher Scientific).

### WMS and data processing

Briefly, purified SCAI-sweep DNA was individually fragmented before library preparation and WMS on a NovaSeq 6000 platform (Illumina, San Diego, USA) with the 150 bp pair-end reads as described [3]. The FASTQ files were subjected to optical duplicates removal, adapter removal, sequence cleaning, human DNA removal and synchronization of unpaired reads as described [3].

### KpSC quantification and taxonomy

Quality controlled (QC) reads were subjected to Kpn abundance quantification and taxonomy profiling. These steps were performed by Kraken2 (version 2.1.2) [21] and Bracken (version 2.6.1) [22], respectively. The Kraken2 default MiniKraken DB_8GB v202003 database was enlarged by the addition of 484 KpSC genomes from the T7 study [17] including Kp1 (*n*=303), Kp2 (*n*=31), Kp4 (*n*=16) and Kp3 (*n*=134), as well as 2109 KpSC genomes from the SpARK study [23] including Kp1 (*n*=427), Kp2 (*n*=62), Kp4 (*n*=38) and Kp3 (*n*=170). Moreover, six non-KpSC *Klebsiella* outliers were added: *Klebsiella aerogenes*, *Klebsiella grimontii*, *Klebsiella huaxiensis*, *Klebsiella michiganensis*, *Klebsiella pasteurii* and *Klebsiella spallanzanii*. We used the Bayesian Gaussian mixture model of PopPUNK version 2.0.2 to cluster the Kp1, Kp2, Kp4 and Kp3 genomes [24]. Furthermore, the python script (extract_kraken_reads.py) from the KrakenTools package [25] was used to extract FASTQ reads assigned to the taxonomy ID: 570 (*Klebsiella* genus) with ‘children’ based on the Kraken2 taxonomy classification output files.

### KpSC ST typing using mSWEEP and StrainGE

We used the mSWEEP (version mSWEEP_linux-v1.5.2) and StrainGE (version 1.3.7) toolkits to analyse the Kpn ST presence and diversity in the described WMS Kpn-positive SCAI sweeps. Briefly, mSWEEP is designed to estimate the relative abundance of bacterial lineages using an extensive reference genome database [18]. mSWEEP clusters the reference genomes in biologically relevant groups, performs read pseudoalignment to the references, infers probabilistic cluster relative abundances and includes a step that controls the false-positive detections [18]. The Strain Genome Search (StrainGE) toolkit has two main components [19], the Strain Genome Search Tool (StrainGST) and the Strain Genome Recovery (StrainGR). Briefly, StrainGST is a k-mer-based tool that indicates the closest reference genome(s) to the strain(s) in a sample. The StrainGR pipeline builds a concatenated reference based on the reference strains reported by StrainGST, aligns reads and analyses alignments to variant calls (SNPs and large deletions) [19]. The StrainGST can be used by itself as a standalone tool to report the presence of closely related genomes to the reference database and their relative abundance.

To identify the *Klebsiella* strain level and ST diversity, the extracted reads obtained from the Kraken2 output were subjected to the mSWEEP default database [18] and a custom StrainGE [19] database consisting of 1,305 Kpn NCBI refseq genomes downloaded on 18 October 2022 using NCBI Genome Downloading Scripts version 0.3.1 (https://github.com/kblin/ncbi-genome-download). Both databases were enriched by KpSC genomes from T7 (*n*=484) and SpARK collection (*n*=2109). ST detection was performed for both methods with a cut-off of 5% relative abundance to decrease possible false-positive Kpn ST-type detections.

### Genomic characterization of Kpn mSWEEP bins

As mSWEEP supports the read binning algorithm by mGEMS, we further evaluated mSWEEP bins for downstream analysis [26]. Specific Kpn ST bins were assembled using SPAdes (version 3.15.5) [27]. Contigs below 1,000 bp and 2× coverage were removed during the assembly to reduce the number of low-quality contigs that might interfere with downstream analysis. Validation of species detection, K-locus, O-locus, AMR and virulence genes was performed on the assembled genomes using Kleborate (version 2.3.2) [28,29].

### Evaluation of StrainGR for phylogenetic relationship analysis

Phylogenetic analysis was performed using the StrainGR pipeline of the StrainGE toolkit. Briefly, StrainGR builds neighbour-joining trees from pairwise distances between strains based on genomic distances calculated from SNP rates via the Jukes–Cantor model. Phylogenetic relationships were constructed between SCAI-sweep WMS samples for three different STs (ST20, ST25 and ST26) which had been detected in at least three samples each (Table 1). A single-colony WGS isolate ST corresponding to one of the WMS samples was used as reference (Table 1).

**Table 1.** Details of WMS SCAI-sweep samples and corresponding single-colony WGS references for phylogenetic relationship analysis of Kpn ST20, ST25 and ST26

Kpn ST20	Kpn ST25	Kpn ST26
SWE14-WMS^*^	SWE46-WMS	SWE26-WMS
SWE36-WMS	SWE35-WMS^*^	SWE20-WMS
SWE72-WMS	SWE45-WMS	SWE23-WMS
–	–	SWE85-WMS^*^
SWE14-WGS reference ST^*^	SWE35-WGS reference ST^*^	SWE85-WGS reference ST^*^

*The corresponding single-colony WGS reference ST and SCAI-sweep WMS samples.

A core SNP alignment was performed for single isolates (ST20) using Snippy (version 4.6.0) (https://github.com/tseemann/snippy). SNP distance matrix was calculated by snp-dists (version 0.8.2) from the core alignment file (https://github.com/tseemann/snp-dists). Maximum likelihood phylogeny of the core genome alignment was constructed using RAxML (RAxML, version 8.2.12) with 1,000 bootstrap [30]. All the phylogenetic trees were visualized by FigTree (version 1.4.4) (http://tree.bio.ed.ac.uk/software/figtree/).

### Statistical analysis

All statistical analysis was performed in R version 4.3.2. Statistical differences between two groups were analysed using a one-sided Wilcoxon signed-rank test (unpaired). Samples in which only a single ST was detected were included to compare concordant and non-concordant groups for mSWEEP and StrainGE and to evaluate the effect of read numbers on mSWEEP performance and p-kmers on StrainGE performance. Analysis of differences between three or more groups was performed by Kruskal–Wallis test followed by Wilcoxon test with Bonferroni correction. Concordance between ST detection by WGS and each metagenomic method (mSWEEP and StrainGE) was quantified using Cohen’s kappa (*κ*), which measures agreement between categorical classifications beyond chance, using the R package *irr* (version 0.84.1). *P*-values ≤0.05 were considered significant.

### Rarefaction analysis

A simulation-based rarefaction analysis was performed to estimate the effective *Klebsiella* genus read sequencing depth required for reliable ST detection. For each sample with a WGS-concordant ST detected, the vector of mSWEEP-assigned read counts to the concordant ST was down-sampled to a series of target *Klebsiella* genus read depths using multinomial sampling. Target depths began at the lowest *Klebsiella* genus read count observed in our sample collection (SWE93) and increased in intervals up to 20 million *Klebsiella* genus reads (175,101; 3×10⁵; 1×10⁶; 2×10⁶; 5×10⁶; 1×10⁷; and 2×10⁷ reads). At each depth, down-sampling was repeated 100 times to account for stochastic variation, and an ST was considered detected if the down-sampled ST-assigned read count exceeded an empirically derived threshold of 1.5×10^5^ reads. This threshold was defined as the lowest number of mSWEEP-assigned reads associated with an ST that was concordant with WGS (SWE71), compared to the highest read count among non-concordant ST detections not attributable to colony picking of a low-abundance ST (SWE8). Rarefaction analysis was conducted using mSWEEP outputs only, due to the high concordance between mSWEEP and StrainGE in both ST detection and agreement with WGS results.

### Computational resources

The computations were performed on resources provided by Sigma2 – the National Infrastructure for High Performance Computing and Data Storage in Norway (https://www.sigma2.no/) (project number: nn9794k).

### Ethics statement

Collection and analysis of samples for this study was approved by the Regional Committee for Medical and Health Research Ethics, North Norway (REC North reference: 2016/1788, 2014/940 and 67006).

## Results

### Detection Kpn ST by mSWEEP and StrainGST in SCAI-sweep metagenomes compared to single-colony WGS

A total of 52 Kpn culture-positive faecal samples underwent SCAI-sweep enrichment WMS, followed by analysis by mSWEEP and the StrainGST component of StrainGE for Kpn ST presence and diversity and compared to ST detection by single-colony WGS. The selected SCAI-sweep samples, total read counts, percentage of Kraken2 extracted *Klebsiella* genus reads, single-colony WGS Kpn-isolate ST results, the WMS Kpn ST-typing results and ST-matching read and k-mer numbers from analysis by mSWEEP and StrainGE, respectively, are given in Table 2. Notably, 49 samples had a single Kpn ST detected by single-colony WGS, whereas three samples (SWEs 21–23) had a Kp1 and Kp3 ST detected within the same sample. Processed WMS samples ranged from 32 to 107×10^6^ PE reads (mean: 62×10^6^; median: 58×10^6^; sd: 22×10^6^) (Table 2). Kraken2 extracted *Klebsiella* (genus) reads varied from 0.3 to 96% relative abundance of sweep metagenomes (mean: 39.2%, median: 28%, sd: 33.2%).

**Table 2.** mSWEEP and StrainGE ST typing compared to single-colony WGS

ID	Total PE reads per sample post-QC	*Klebsiella* genus PE reads (%)^‡^	T7 single colony	mSWEEP					StrainGE					
				*Klebsiella* identified (cut-off 5%)		(%) ^§^	No of PE reads assigned to the ST type	Concordance with T7 single colony	*Klebsiella* identified (cut-off 5%)		(%) ^§^	pkmers ||	score ^¶^	Concordance with T7 single colony
SWE1*	46320236	27.6	Kp1 ST4633	Kp1	ST4633	100	12435560	yes	Kp1	ST4633	96.8	70933	0.989	yes
SWE2*	50571824	86.2	Kp1 ST3043	Kp1	ST3043	95.9	42270436	yes	Kp1	ST3043	96.1	102087	0.99	yes
SWE3*	42090900	28.1	Kp1 ST1750	Kp1	ST1750	92.9	10045474	yes	Kp1	ST1750	82.8	139981	0.77	yes
				Kp4	ST4096	0.7	559500	–	Kp3	ST4608	7.4		0.298	–
				–		–	–	–	K4	ST4096	6.3		0.355	–
SWE7*	40182364	20.3	Kp1 ST1862	Kp1	ST1862	80.4	6341806	Yes	Kp1	ST1862	47.7	102166	0.833	yes
				Kp4	ST1308	12	1784194	–	Kp4	ST1308	48.2		0.552	–
				Kp4	ST480-2LV	7.6	1763298	–	–		–		–	–
SWE8*	40894246	6.1	Kp1 ST14	Kp4	ST4096	99.6	2408212	No	Kp4	ST4096	94.3	57059	0.976	no
SWE11*	86612976	0.9	Kp1 ST4255	nd		–	22942	No	nd		–	8296	–	no
SWE12*	105237936	59.5	Kp1 ST70	Kp1	ST70	100	60290028	Yes	Kp1	ST70	93.9	121728	0.992	yes
SWE13*	99518122	34.7	Kp1 ST45	Kp1	ST45	100	33881356	Yes	Kp1	ST45	96.8	92245	0.887	yes
SWE14*	106158610	92.8	Kp1 ST20	Kp1	ST20	72.6	70926504	Yes	Kp1	ST20	95.8	128854	0.992	yes
				Kp1	ST5100	27.4	26981624	–	–		–		–	–
SWE18*	97761156	27.1	Kp1 ST4039	Kp1	ST4039	99.7	25525020	Yes	Kp1	ST4039	95.7	86471	0.984	yes
SWE19*	96593632	24.8	Kp1 ST17	Kp1	ST17	92	21819079	Yes	Kp1	ST17	83.8	94281	0.944	yes
				Kp1	ST16	7.8	1867809	–	Kp1	ST1428	6		0.042	–
SWE20*	107490984	8.2	Kp1 ST26	Kp1	ST26	93	7465576	Yes	Kp1	ST26	82.9	68390	0.91	yes
SWE21*	101125792	47	Kp1 ST151	Kp1	ST151	91.9	26559926	Yes	Kp1	ST151	50.6	157363	0.871	yes
			Kp3 ST1209-3LV	nd		–	2808576	No	Kp3	ST355	9.4		0.345	No
				–		–	–	–	Kp3	ST1562	6.2		0.05	No
SWE22*	101959022	2.8	Kp1 ST27	Kp3	ST3084	63.4	1690910	Yes	Kp3	ST3084	57.2	98873	0.611	Yes
			Kp3 3084	Kp1	ST27	34.6	979378	yes	Kp1	ST27	32.3		0.707	Yes
SWE23*	94301508	9.5	Kp1 ST26	Kp3	ST681	47.8	3761724	Yes	Kp1	ST26	50	109651	0.621	Yes
			Kp3 ST681	Kp1	ST26	42.6	3463628	Yes	Kp3	ST681	44.2		0.776	Yes
SWE34^†^	43039094	42.2	Kp1 ST157	Kp1	ST157	100	16855580	Yes	Kp1	ST157	93.6	84573	0.988	Yes
SWE35^†^	44126160	44.6	Kp1 ST25	Kp1	ST10	84.3	15888556	–	Kp1	ST10	78.7	104240	0.932	–
				Kp1	ST25	13.2	7214704	Yes	Kp1	ST25	12		0.525	Yes
SWE36^†^	57118608	68.1	Kp1 ST20	Kp1	ST20	100	37630002	Yes	Kp1	ST20	94.4	109208	0.989	Yes
SWE40^†^	42285706	6.4	Kp1 ST4649	Kp1	ST4649	96.5	2387544	Yes	Kp1	ST4649	88.1	67195	0.951	Yes
				Kp1	ST3164	5.4	10618180	–	Kp1	ST1565	29.3		0.139	–
SWE41^†^	32664222	88.4	Kp1 ST405	Kp1	ST405	100	26725162	Yes	Kp1	ST405	95	97582	0.992	Yes
SWE42	42938740	26.2	Kp1 ST4248	Kp1	ST4248	72.3	7280928	Yes	Kp1	ST4248	69	85974	0.906	Yes
SWE43^†^	43039094	18.5	Kp1 ST4643	Kp1	ST4643	100	7481886	Yes	Kp1	ST4643	94.4	70385	0.96	Yes
SWE44^†^	55848022	74.7	Kp1 ST23	Kp1	ST23	100	40507736	Yes	Kp1	ST23	48.6	109471	0.928	Yes
				–		–	–	–	Kp1	ST1660	47		0.153	–
SWE45^†^	51827146	59.7	Kp1 ST25	Kp1	ST25	86.2	23336270	Yes	Kp1	ST25	57.5	121952	0.938	Yes
				Kp1	ST3164	5.4	10618180	–	Kp1	ST1565	29.3		0.139	–
SWE46^†^	37854950	87.9	Kp1 ST25	Kp1	ST461	60.2	21880254	–	Kp1	ST461	27.9	119817	0.832	–
				Kp1	ST25	34.6	16104768	Yes	Kp1	ST25	18.5		0.413	Yes
SWE47^†^	77698532	9.9	Kp1 ST11	Kp1	ST11	95.6	4850910	Yes	Kp1	ST11	65.9	75996	0.91	Yes
SWE70^†^	69417118	4.3	Kp1 ST37	Kp1	ST37	63.7	1722882	Yes	Kp1	ST37	90.2	62521	0.971	Yes
				Kp1	ST256	21.3	2247810	–	–		–		–	–
				Kp1	ST2370	11.3	2232222	–	–		–		–	–
SWE71^†^	50416960	0.6	Kp1 ST2248	Kp1	ST2248	70.3	150136	Yes	Kp1	ST2248	52.9	54645	0.796	Yes
SWE72^†^	57398656	28.8	Kp1 ST20	Kp1	ST20	97.8	12432384	Yes	Kp1	ST20	71.9	93006	0.947	Yes
SWE73^†^	53382614	55.9	Kp1 ST10	Kp1	ST10	100	28641772	Yes	Kp1	ST10	91.9	99970	0.908	Yes
SWE74^†^	40635788	92.4	Kp1 ST27	Kp1	ST27	100	36190262	Yes	Kp1	ST27	94.5	98679	0.989	Yes
SWE75^†^	62898100	96	Kp1 ST485	Kp1	ST485	93.8	50181476	Yes	Kp1	ST485	81.3	143314	0.909	Yes
				Kp1	ST35	6.2	20176438	–	Kp1	ST35	13.1		0.444	–
SWE84*	62548172	1.4	Kp1 ST200	Kp1	ST200	90.1	694590	yes	Kp1	ST200	76.2	54800	0.973	Yes
SWE85*	62401154	8.4	Kp1 ST26	Kp1	ST26	74.9	3934072	Yes	Kp1	ST26	44.7	82744	0.897	Yes
				Kp1	ST2791	15.9	1912382	–	Kp1	ST27	40.2		0.384	–
				Kp1	ST27	8.6	1625020	–	Kp1	ST2791	9.6		0.059	–
SWE86*	64269478	95.7	Kp1 ST515	Kp1	ST515	100	60569230	Yes	Kp1	ST515	97	100861	0.989	Yes
SWE87*	66225340	1.8	Kp1 ST4658	Kp1	ST4658	89.2	1039800	Yes	Kp1	ST4658	85.2	59624	0.922	Yes
SWE88*	49928990	7.4	Kp1 ST37	Kp1	ST37	71.1	1407258	Yes	Kp1	ST37	86.3	59010	0.964	Yes
				Kp1	ST2370	16.1	2612108	–	–		–		–	–
				Kp1	ST256	12.8	2607288	–	–		–		–	–
SWE89*	58437944	76.5	Kp1 ST14	Kp1	ST14	100	44014250	Yes	Kp1	ST14	97.6	96020	0.987	Yes
SWE90*	56103864	44.3	Kp1 ST1106	Kp1	ST1106	31.3	7787674	Yes	Kp2	ST1681	66.2	137705	0.53	–
				Kp2	ST1681	6.3	6433926	–	Kp1	ST1106	25.1		0.417	Yes
				Kp1	ST449	5.4	46277106	–	–		–		–	–
SWE91*	63984542	93.9	Kp1 ST1496	Kp1	ST1496	100	59207556	Yes	Kp1	ST1496	97.8	104627	0.986	Yes
SWE92*	54636568	91	Kp1 ST35	Kp1	ST35	94.6	42732572	Yes	Kp1	ST35	97.8	97993	0.983	Yes
				Kp1	ST449	5.4	46277106	–	–		–		–	–
SWE93*	58366906	0.3	Kp1 ST20	Kp1	ST159	49.5	73494	No	nd		–	3981	–	No
SWE94*	61225402	8.8	Kp1 ST704	Kp1	ST704	96.7	5146204	Yes	Kp1	ST704	96.3	64242	0.949	Yes
SWE95*	61898468	20.1	Kp1 ST4632	Kp1	ST4632	67	8478160	Yes	Kp1	ST4632	57.2	87724	0.881	Yes
SWE96*	58234202	47.1	Kp1 ST1145	Kp1	ST1145	100	26859338	Yes	Kp1	ST1145	97	79918	0.991	Yes
SWE97*	67057622	80.6	Kp1 ST704	Kp1	ST704	96.5	52001674	yes	Kp1	ST704	77.3	120447	0.985	Yes
				–		–	–	–	Kp1	ST253	19		0.038	–
SWE98*	57525522	8.3	Kp1 ST4657	Kp1	ST4657	73.7	4241620	Yes	nd		84.7	74444	0.902	No
				Kp1	ST584	11.9	3802654	–	Kp1	ST584	7		0.232	–
				Kp1	ST1832	7.8	1589170	–	Kp1	ST1832	4.7		0.102	–
				Kp1	ST2004	6.6	3346678	–	Kp1	ST2004	–		–	–
SWE99*	61137200	2.3	Kp1 ST461	Kp1	ST461	74.7	1060320	Yes	Kp1	ST461	93.2	59455	0.937	Yes
				Kp1	ST3660	22.3	1179836	–	–		–		–	–
SWE100*	55978856	27.9	Kp1 ST375	Kp1	ST375	99.7	14979132	Yes	Kp1	ST375	95.5	74785	0.972	Yes
SWE101*	59994.99	15.6	Kp1 ST2042	Kp1	ST2042	77.9	9101702	Yes	Kp1	ST2042	97	71886	0.965	Yes
				Kp1	ST4660	22.1	9022308	–	–		–		–	–
SWE102*	66525008	86.3	Kp1 ST2039	Kp1	ST2039	100	56193170	Yes	Kp1	ST2039	96.5	102765	0.992	Yes
SWE103*	71616674	41.7	Kp1 ST4660	Kp1	ST4660	74.2	29058728	Yes	Kp1	ST2042	94.4	93507	0.976	No
				Kp1	ST2042	25.8	28862122	–	–		–		–	–

*Samples sequenced in Japan.

†Samples sequenced in Oslo/Norway.

‡Total number of *Klebsiella* genus PE reads extracted from Kraken2.

§Per cent of reads assigned to the ST.

||Total unique number of k-mers presented in the database.

¶Confidence score in the prediction.

The overall concordance in Kpn ST detection between mSWEEP and StrainGST compared to the single-colony WGS is presented in Table 3. Briefly, mSWEEP-detected STs were concordant with the reference single-colony WGS ST in 46/49 single-ST and 2/3 double-ST samples, respectively. The corresponding results for StrainGST were 44/49 and 2/3, respectively. This gave very strong agreement in ST detection with WGS for both methods, with Cohen’s *κ* 0.93 (*P*<0.001) for mSWEEP and 0.89 (*P*<0.001) for StrainGE, respectively.

**Table 3.** Concordance in mSWEEP and StrainGST WMS ST-results from SCAI-sweep samples compared to single-ST (*n*=49) and double-ST (*n*=3) single-colony WGS STs

WMS samples with concordance (+) and discrepancies (-) between mSWEEP and StrainGST ST results, respectively, compared to the single-colony WGS ST				
**Concordance with WGS**	**+/+**	**-/+**	**+/-**	**-/-**
**Single ST (*n*=49**)	44	0	2	3
**Double ST (*n*=3)**	2	0	0	1

Discrepancies to single-colony WGS STs were observed in samples SWE8, SWE11 and SWE93 in both mSWEEP and StrainGST metagenome analysis. Both mSWEEP and StrainGST detected the same alternative and dominant ST (ST4096) in SWE8 compared to the single-colony WGS-detected ST14, suggesting that the colony picking for WGS analysis had selected a minority ST in this sample. In SWE93, mSWEEP analysis detected an alternative ST (ST159 vs single-colony WGS ST20), whereas no Kpn-ST was identified by StrainGST, suggesting that this was not due to the detection of low-abundance ST by the colony picking strategy. No ST was detected by either mSWEEP or StrainGE in SWE11 (single-colony WGS ST4255). A discrepancy to single-colony WGS ST was also observed in SWE103 in StrainGST analysis only (ST2042 vs single-colony WGS ST4660). In this sample, StrainGST identified an alternative ST (ST2042) which was also identified by mSWEEP as the second most abundant Kpn-ST and is likely a true-positive finding. Similarly, StrainGE did not detect the single-colony WGS-detected ST4657 in SWE98; however, it detected three additional STs which were also detected by mSWEEP.

Regarding sweep samples with two detected STs from single-colony WGS (SWE21-23), both STs were identified by mSWEEP and StrainGST in 2/3 samples (SWE22 and -23). Only one of the WGS-detected STs (ST151) was detected by both mSWEEP and StrainGST in SWE21. The secondary WGS-detected ST in SWE21, ST1209-3LV (Kp3 phylogroup), was not detected by either mSWEEP or StrainGST. In this sample, two alternative Kp3 STs were detected by StrainGST (ST355 and ST1562), while no additional STs were detected by mSWEEP.

The relative abundance of *Klebsiella* genus reads estimated by Kraken2 in sweep samples with concordant results to single-colony WGS ranged from 0.6 to 96 % (mean: 41.5%, median: 28.8% and SD: 32.9%). Despite sample SWE71 having the second lowest abundance of *Klebsiella* genus reads of all sweep samples (0.6%), this sample was still concordant with the single-colony WGS ST. The Kraken2 estimated *Klebsiella* genus abundances in sweep samples that were non-concordant with single-colony WGS by both mSWEEP and StrainGST (SWE8, -11 and -93) were low (6.1%, 0.9% and 0.3% respectively).

**Fig. 1.**
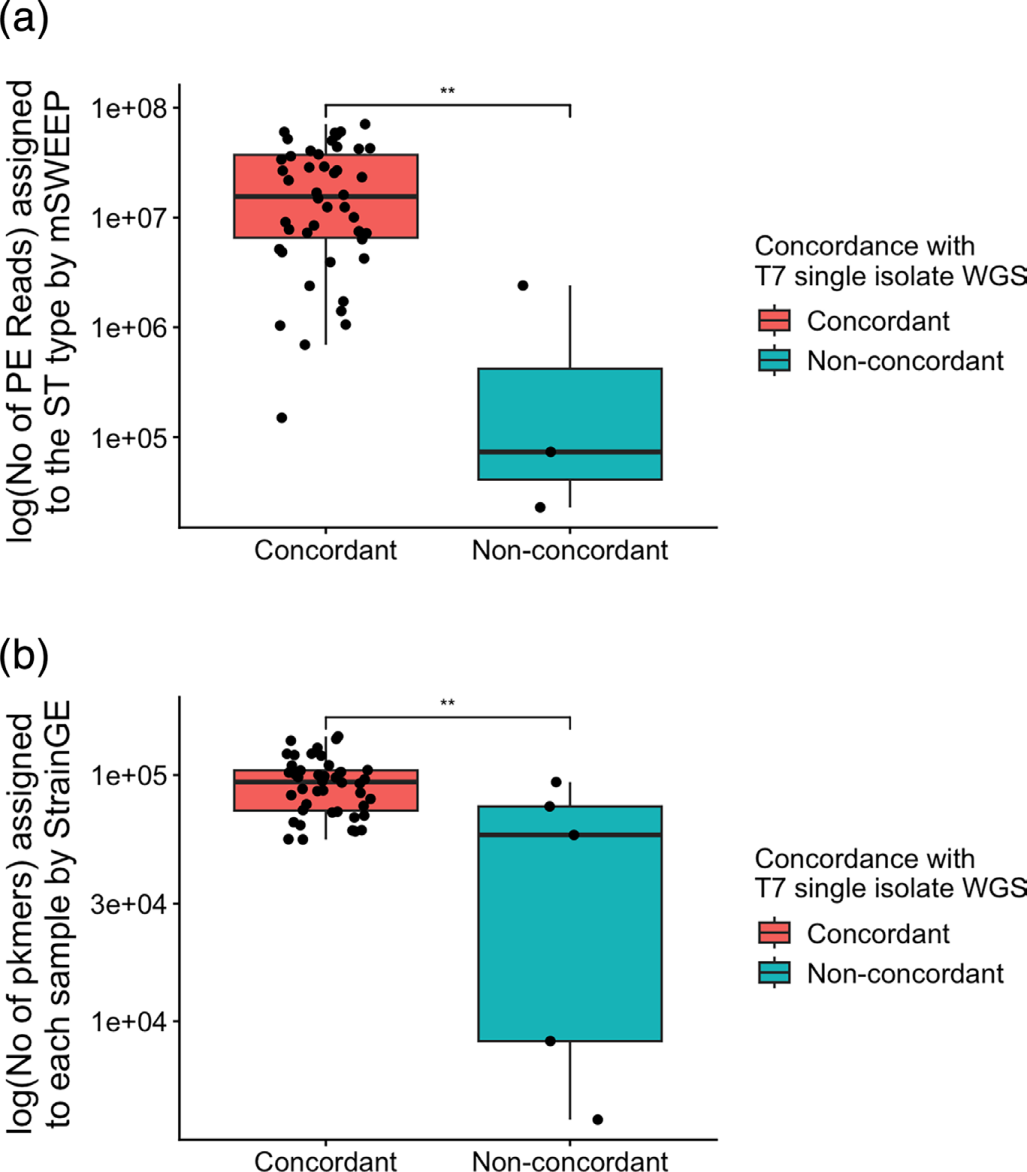
Comparison of (a) read numbers assigned to STs by mSWEEP and (b) k-mers in samples assigned to the KpSC database by StrainGST (pk-mers) between samples with concordant and non-concordant STs to single-colony WGS ST detection. **P<0.01 (single-tailed Wilcoxon signed-rank test).

We next investigated whether the observed non-concordance of the STs between single-colony WGS detection and mSWEEP/StrainGE was related to a lower number of reads assigned to STs by these tools. Comparing the number of reads assigned by mSWEEP between concordant and non-concordant STs demonstrated that concordant STs had a significantly higher number of assigned reads than the non-concordant STs (*P*=0.002) (Fig. 1a). Similarly, comparing the total number of unique k-mers in samples that matched the database (pk-mers) between concordant and non-concordant samples by StrainGST demonstrated that concordant samples had significantly higher number of k-mers than the non-concordant samples (*P*=0.006) (Fig. 1b) .

### Detection of within-sample ST diversity

Within-sample Kpn ST diversity (≥2 STs) was detected in 19 and 15 samples by mSWEEP and StrainGST analysis, respectively (Table 2). Twelve sweep metagenomes with multiple STs identified overlapped between mSWEEP and StrainGST, of which the same additional STs were identified by both tools in ten samples. The maximum number of STs identified per sample was four detected by mSWEEP in sample SWE98 (Table 2).

### Estimation of minimum required genome coverage and rarefaction analysis

Minimum genome coverage for reliable ST detection was estimated from sample SWE71, as this sample had the lowest number of mSWEEP assigned reads and StrainGE assigned pk-mers that resulted in ST detection concordant with WGS (*n*=150,136 reads; Table 2). Assuming an average genome size of 5.5 Mbp, this equated to ~4.1× genome coverage at 150 bp reads [31]. Compared to sample SWE93, which had the highest number of mSWEEP assigned reads to a non-concordant ST that was not detected by StrainGE, thus could not be explained by colony picking of a low-abundance ST (*n*=73,494 reads; Table 2), this approximated to 2.0× genome coverage. Thus, under the experimental conditions used, we consider a threshold of ~150,000 assigned reads – corresponding to ~4× genome coverage – to be a practical minimum requirement for reliable ST detection.

**Fig. 2.**
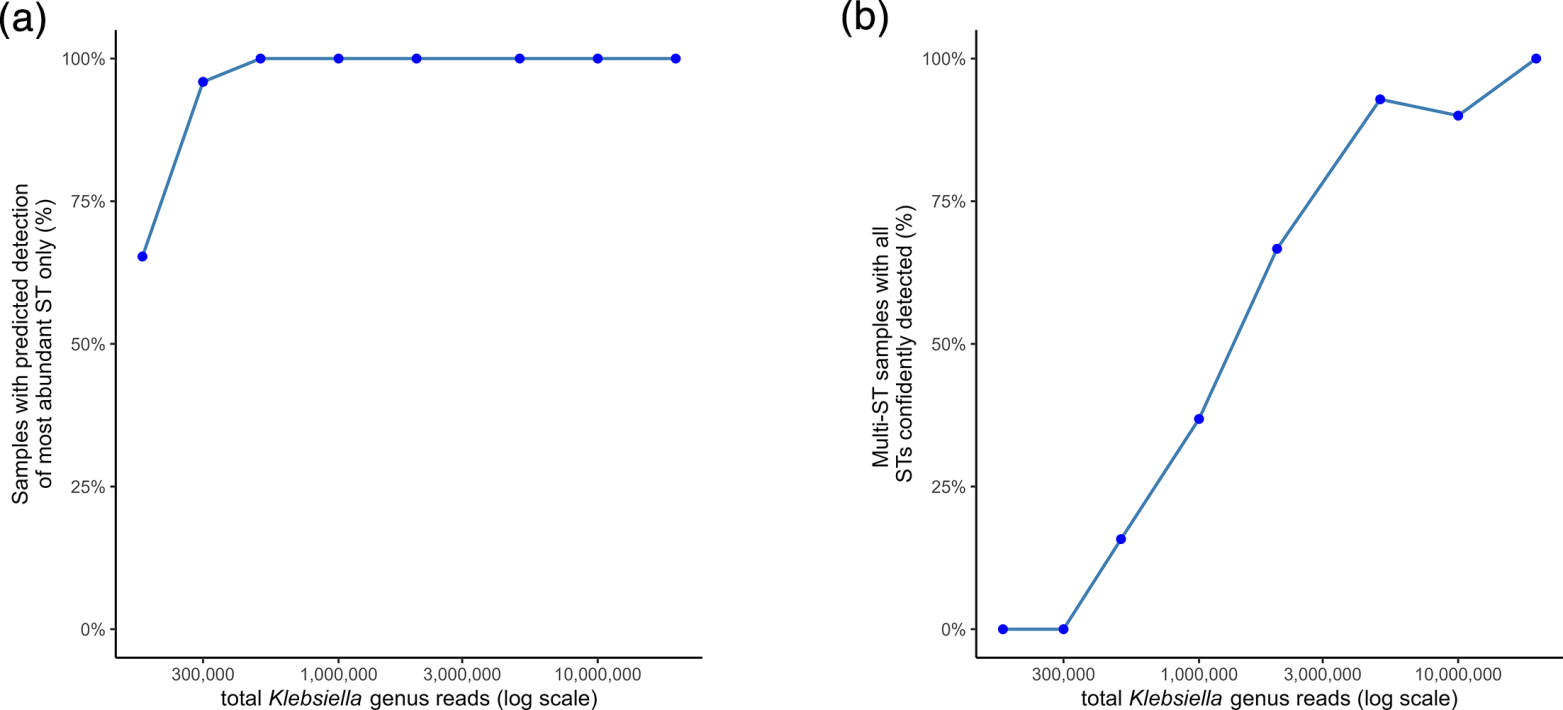
Rarefaction analysis estimating *Klebsiella* genus read depth required for reliable ST detection. (a) Percentage of samples in which detection of the dominant ST would be predicted to occur at varying Klebsiella genus read depths. (b) Percentage of samples with multiple STs detected in which all STs were predicted to be detected at varying Klebsiella genus read depths. The threshold for confident ST detection (*n*=150,000 ST-assigned reads by mSWEEP) was derived from the sample with WGS concordant ST detection that had the lowest observed Klebsiella genus reads (*n*=150,136, Table 2), compared to the sample with the highest observed *Klebsiella* genus reads that resulted in non-concordant ST detection (*n*=73,494, Table 2).

Using this threshold, we performed rarefaction analysis on all samples with a concordant ST detected by mSWEEP (*n*=49) to investigate the influence of sequencing depth on detection of concordant STs as well as within-sample ST diversity. Rarefaction was performed on the total number of reads assigned to the *Klebsiella* genus, as this was the input to both mSWEEP and StrainGE, and showed large variation between samples (median: 1.73×10^7^ reads, range: 1.73×10^5^–9.85×10^7^; Table 2). When samples were rarefied to the lowest number of *Klebsiella* genus reads observed across our dataset (SWE93; *n*=175,101 reads; Table 2), this analysis predicted that mSWEEP would have detected the most dominant ST only 65.3% of samples (*n*=32/49). In all cases, this would have corresponded to the concordant ST detected by WGS. Detection of at least one ST in 100% of samples, however, was not predicted until there were at least 500,000 *Klebsiella* genus reads (Fig. 2a). Similarly, of the samples with multiple STs detected by mSWEEP (*n*=19), rarefaction analysis predicted that even at depths of up to 1×10^6^ *Klebsiella* genus reads, mSWEEP would have failed to detect more than one ST in 63.2% of samples, and detection of full ST diversity in 100% of samples would occur only at depths above 20×10^6^ *Klebsiella* genus reads (Fig. 2b).

### Comparison of computational resources

We also compared computational resources required for ST detection and abundance by mSWEEP and StrainGE. In our computational setup, mSWEEP required on average 11 h per sample (number of tasks: 1, CPUs per task: 15, memory per CPUs: 66 GB), while StrainGE required on average 1 h per sample (number of tasks: 4, CPUs per task: 8, memory per CPUs: 38 GB). Additionally, while mSWEEP ST analysis required access to high-performance computing, StrainGE ST analysis could be run on a laptop.

### Downstream analysis of the mSWEEP bins

The assembled Kpn binned reads from mSWEEP were further analysed for species, STs, virulence genes, K-locus, O-locus and AMR genes using the Kleborate/Kaptive pipeline (Table S1, available in the online Supplementary Material). We observed species match in 47 assembled genomes with single-colony WGS reference genomes. The five exceptions included SWE-8 (no KpSC match) as well as SWE-11, -71, -90 and -93 (KpSC not detected). We observed 100% concordance in STs between assembled and single-colony WGS genome for the remaining 47 assemblies. Assembly analysis of two samples (SWE-14 and −19) with two STs detected by mSWEEP revealed only one of the reference STs (ST20) in SWE-14 and the reference ST (ST17) plus a single-locus variant of the reference ST (ST17-1LV) in SWE-19.

We also compared virulence genes, K-locus, O-locus and AMR genes between the assembled genomes and single-colony WGS reference genomes for samples that matched STs with corresponding reference STs. Virulence score differed in nine assembled genomes (SWE-34, -47, -72, -92, -94, -100, -101, -102 and -103). Most of the virulence profile matched with a few exceptions: virulence factors were not detected either in reference (yersiniabactin: SWE-44) or in the assembled genome (yersiniabactin: SWE-13, -21, -35, -46, -47, -70, -92 and -94; colibactin: SWE-101, -102 and -103; aerobactin and salmochelin: SWE-100). Hypermucoviscosity *rmp* gene profiles matched across all assembled Kpn bins and reference genomes. K-locus was consistent in 45 of the assembled genomes with references (no match for SWE-42 and -95). We observed differences in O-locus for three samples (SWE-40, -75 and -99).

Overall, we observed no differences in Kleborate resistance scores between the assembled bins and their corresponding single-colony WGS reference genomes. Acquired fluoroquinolone resistance (Flq_acquired) gene profiles matched for all the assembled bins with their reference genome. However, acquired aminoglycoside resistance genes (AGly_acquired) were undetected in SWE-35, -40, -45 and -47 assembled bins but detected in their corresponding reference genomes. Similarly, there were no mismatches in detectable acquired resistance genes against sulphonamide (Sul_acquired), tetracycline (Tet_acquired) and trimethoprim (Tmt_acquired), but several bin assemblies lacked detection (Sul_acquired: SWE-35, -40, -43, -45 and -46; Tet_acquired: SWE-35, -40, -45 and -46; and Tmt_acquired: SWE-35 and -40). Acquired *β*-lactamase genes (Bla_acquired) and SHV mutation profiles also matched very well between assembled and reference genomes with few exceptions (Bla_acquired: SWE-47, -84 and -88; SHV_mutations: SWE-47 and -88).

Assembly metrics (total genome length and N50) did not show any significant differences (*P*>0.4) in total assembly length between genomes that matched features with single-colony WGS reference genomes (median=5,700,622, IQR=5,563,627–6,390,470), genomes that had mismatch features with single-colony WGS reference genomes (median=6,304,149, IQR=5,902,887.75–7,408,573.5) and genomes where the features were undetected/unknown (median=6,032,838.5, IQR=5,411,485.75–7,035,193). But there was a significant reduction in N50 values (*P*=0.035) of assembled genomes where some features were undetected/unknown (median=101,011, IQR=15,815.5–150,611.25) than the genomes where all the features matched with reference genomes (median=159,384, IQR=92,876–180,565).

### Evaluation of StrainGR in analysing phylogenetic relationships between identical Kpn STs from different metagenome samples

To investigate the ability of the StrainGR component of StrainGE to accurately analyse relationships between closely related Kpn strains, phylogenetic analysis was performed on all sweep samples in which the same ST had been identified [ST20 (*n*=3 samples), ST25 (*n*=3 samples) and ST26 (*n*=4 samples)], compared to a single-colony WGS isolate from one of the samples as reference (Table 1). Phylogenetic analyses of ST20 samples (SWE14, SWE36 and SWE72) confirmed that ST20 detected in the SWE14 sweep metagenome was the same strain as the corresponding single-colony WGS isolate SWE14 ST20, localizing within the same clade with no estimated genetic distance between these two STs (Fig. S1, Table S2a). The SWE36 and SWE72 WMS-detected ST20 strains belonged to a different clade than both the SWE14 WMS-detected ST20 strain and corresponding single-colony WGS ST20 strain (Fig. S1). Similarly, analysis of the ST26-containing sweep samples (SWE20, SWE26, SWE23 and SWE85) confirmed the presence of SWE85 WMS-detected ST26 and corresponding single-colony WGS ST26 in the same clade, although estimated a genomic distance of 0.000001 between these strains (Fig. S2, Table S2c). The SWE20, SWE26 and SWE23 were more distantly related (Fig. S2, Table S2c). Analysis of ST25 samples (SWE46, SWE45 and SWE35), however, demonstrated the presence of SWE35 WMS-detected and single-colony WGS ST25 strains in different clades with estimated genomic distance of 0.00001 (Fig. S3, Table S2d). Notably, this ST25 was identified as the minority ST in this sweep sample, which was dominated by ST10, with relative abundances 12 and 78.7% respectively. Moreover, the unique k-mers assigned to ST25 in this sweep sample (*n*=11,042) were considerably lower than the average unique k-mers assigned to all STs in the phylogenetic analysis (*n*=47,641). The single-colony WGS SWE35 ST25 strain was placed in the same clade as the SWE46 WMS-detected ST25 despite not corresponding to this sweep sample (Fig. S3). The SWE45 WMS-detected ST25 was assigned to a different clade than the other three ST25 strains (Fig. S3).

The accuracy of the phylogenetic relationships between the WMS-detected ST20 strains created by StrainGR was also compared to the core genome phylogeny of the corresponding ST20 isolates from these samples. The core genome phylogeny of ST20 isolates demonstrated similar phylogeny structures compared to that of ST20 metagenome samples created by StrainGR (Fig. S4, Table S2b). As was identified by StrainGR, the single-colony WGS STs from SWE36 and SWE72 were more closely related to each other than single-colony isolate from SWE14, depicted from the location of the clades (Fig. S4). The ST25 SWE35 single-colony WGS isolate, which was included as a distantly related outlier, was located furthest away in the tree. The pairwise SNP distance matrices of the ST20 isolates also had similar relative values to the corresponding pairwise estimated genomic distances between WMS-detected ST20 strains (Table S2a, b). Thus, phylogenetic relationships of the ST20 single isolates using core genome alignment corresponded to the phylogenetic relationships of the WMS-detected ST20 strains predicted by StrainGR.

## Discussion

The global spread of AMR by Kpn is tightly linked to the clonal dissemination of a relatively small number of high-risk lineages [5,32]. Moreover, HyV in Kpn is strongly associated with lineages within CG23 [33]. The ability to accurately detect and analyse Kpn at the subspecies level is therefore essential for public health surveillance and to better understand Kpn colonization dynamics and ecology. Here, we have taken advantage of our previous study of SCAI-sweep faecal samples with identified Kp1 and Kp3 STs using single-colony WGS [3,17] to evaluate and compare the performance of two newly developed WMS-based bioinformatic tools to identify Kpn ST diversity in culture enriched bacterial metagenome samples.

Both the mSWEEP and StrainGST analysis of culture-enriched SCAI-sweep metagenomes showed a high concordance with the expected Kpn STs from the previous single-colony WGS data [17]. The non-concordance observed in STs was directly proportional to the lower number of PE reads and k-mers assigned by mSWEEP and StrainGE, respectively. Moreover, the agreement between mSWEEP and StrainGE in alternate STs detected in non-concordant samples SWE8, SWE98 and SWE103 suggests that these were true STs present in these samples that were not detected by WGS due to the single-colony picking strategy used.

Our findings further indicate the importance of sufficient read coverage in making accurate ST identification by these tools. The minimum coverage required for accurate strain identification by mSWEEP has been reported as ~0.30× for Kpn, corresponding to at least 16,000 reads from a specific lineage in a dataset of 1,000,000 reads (100 bp read length) [18]. Similarly, StrainGE reports as low as 0.1 × coverage for accurate strain detection [19]. Based on our results, under the experimental conditions used, we estimated that sufficient read coverage to ~4×, corresponding to approximately 150,000 ST-specific reads (150 bp read length). This increased requirement may have been related to the SCAI-culture enriched faecal samples retaining a complex assortment of closely related Enterobacterales, as well as the high diversity and close relatedness of the KpSC phylogeny itself, making reliable differentiation between STs challenging [20,31]. Indeed, rarefaction analysis predicted that if all samples had received the same number of *Klebsiella* genus reads equal to that of the lowest sample, mSWEEP would have only detected 65.3% of concordant STs. Similarly, detection of the full within-sample ST diversity achieved by mSWEEP was not predicted to occur until the *Klebsiella* genus reached 20×10^6^ reads. As only 46% of all samples (*n*=24/52) reached this depth, it is therefore possible that our analysis may have underestimated true Kpn ST diversity of our samples.

In the three samples with two STs detected by single-colony WGS, both mSWEEP and StrainGST correctly identified both STs in two samples and one of the two STs in the third sample. Furthermore, we observed good concordance between mSWEEP and StrainGST in detection of within-sample Kpn ST diversity in 10 out of 12 samples. According to the nutrient-niche hypothesis, closely related strains and species are potentially important competitors within the gut microbiome due to large overlap in metabolic requirements [34]. Indeed, *Klebsiella oxytoca* has been suggested to be an important inhibitor of Kpn within the gut microbiome *in vivo* [35]. Within-sample Kpn strain diversity and co-carriage of Kpn with other *Klebsiella* genera, however, has been previously reported [36,38]. Moreover, horizontal gene transfer (HGT) of carbapenemase genes between Kpn strains has been observed within a co-colonized gut microbiome [39]. Studying co-colonization of multiple Kpn strains within a single gut microbiome is therefore important to better understand within-species competition and cooperation, and the extent and conditions under which HGT occurs in this setting. The ability of WMS-based tools to accurately detect and study within-sample Kpn ST diversity is therefore an important advantage of this technology over single-colony WGS, in which such detection is more laborious.

Genomic analysis beyond ST detection, such as identifying determinants of AMR and virulence, has significant impact on treatment decisions and infection outcomes. mSWEEP is designed for metagenomic binning which allows for genome assembly-based downstream analysis of such determinants. However, it is important to firstly evaluate the reliability of Kpn read-binning by mSWEEP in order to perform accurate downstream analysis. The majority of genomes assembled from the mSWEEP bins reported the same species as the single-colony WGS reference genome with only two mismatches. Although there was no discrepancy in detected STs between mSWEEP-assembled genomes and the corresponding single-colony WGS reference genomes, we found additional STs detected from direct mSWEEP analysis in two samples that did not match either the mSWEEP-assembled genomes or the single-colony WGS reference. These additional STs differed from the mSWEEP-assembled genomes by only a single allele, suggesting that direct mSWEEP detection had assigned a single strain to two very closely related genomes in its database. These findings indicate that ST assignment based on assembly of the mSWEEP bins gives better resolution of ST detection compared to direct mSWEEP analysis. We also screened the assembled Kpn genomes from mSWEEP bins for Kpn serotype as well as AMR and virulence determinants and compared these with single-colony WGS reference genomes. Most of the assembled genomes had the same K-locus, *wzi* allele, O-locus and yersiniabactin as the single-colony WGS reference genome. Most of the virulence and resistance profiles of the assembled genomes also matched the corresponding reference genomes. Assembly metrics indicated that fragmentation or lack of contiguity measured by reduced N50 might affect successful downstream analysis. These suggest that mSWEEP bins can be useful for downstream analysis; however, data generated from mSWEEP bins needs to be evaluated carefully to prevent misinterpretation.

During clonal outbreaks, it is crucial to identify infection sources and characterize transmission dynamics to support targeted containment efforts [40]. Phylogenetic analysis reconstructs the evolutionary relationships between strains, which helps to track transmission between individuals or communities and identify the sources of infection or reservoirs [41]. StrainGR phylogenetic analysis showed that, for two of the three STs tested (ST20 and ST26), isolate and sweep samples from the same individual were more closely related than sweep samples of the same ST from different individuals. Core genome alignment of ST20 isolates also validated this finding. However, StrainGR failed to identify the single-colony WGS ST25 isolate from sample SWE35, and the corresponding WMS-detected ST25 was in fact the same strain. ST25 in this sample was detected as a minority ST with comparatively lower k-mer coverage by StrainGST. This indicated that the accuracy of phylogenetic relationships predicted by StrainGR may be compromised at lower coverage levels. Although our findings demonstrate that StrainGR shows promise in recreating phylogenetic relationships between closely related Kpn strains, further studies involving a larger number of samples containing closely related strains are required to better define the accuracy and limitations of StrainGR in this setting.

Both tools performed well in terms of identifying the Kpn ST that corresponded to single-colony WGS detection. mSWEEP required more CPU hours and more computing resources compared with StrainGST, which was related to the binning process of mSWEEP. Despite this, the ability to bin reads is an important advantage of mSWEEP, allowing recovery and assembly of strain genomes for in-depth downstream analysis. While StrainGE does not have this function, it nonetheless demonstrated the ability to create accurate phylogenetic relationships between closely related strains. In addition, within the StrainGR component of StrainGE is also the ability to perform strain-aware variant calling at low coverages which was not explored as part of this study [19]. Both algorithms have no limitations considering the input size (sample size). Outputs for both tools were easily interpreted. Overall, this study suggests the preferred use of mSWEEP when the research goal involves reconstructing or characterizing complete strain genomes from metagenomic data. In contrast, StrainGE is more suitable for rapid and computationally efficient detection of strain-level diversity in large datasets. Both tools produce interpretable outputs and can be effectively applied depending on the specific analytical objective and available computational resources.

The limitations of the study were the low number of samples examined and the limited number of WGS single colonies picked per sample for comparison to sweep WMS detection. Additionally, the reference database included Kpn genomes isolated from the same faecal sweep samples used in the analysis, which introduces the possibility of detection bias. However, both tools had access to the same reference database which also had unique STs from the baseline T7 study making the analysis comparable [17].

## Conclusions

This study demonstrates the potential of WMS-based tools to perform accurate sub-species detection of Kpn in complex microbial communities. While StrainGE and mSWEEP both accurately detected STs when compared to single-colony WGS, both tools had differing strengths and weaknesses. These were predominantly related to differences in computing requirements and potential for downstream genome recovery and analysis. The ability to accurately detect within-sample ST diversity was a strength of both StrainGE and mSWEEP. Utilizing these tools in future studies can enhance our understanding of Kpn colonization ecology and dynamics, with potential to inform future public health surveillance and high-risk Kpn strain tracking.

## Supplementary material

10.1099/mgen.0.001638 Uncited Supplementary Material 1.

## Abbreviations

AMR: antimicrobial resistance
CGs: clonal groups
HGT: horizontal gene transfer
HyV: hypervirulence
Kpn: *Klebsiella pneumoniae*
KpSC: *Klebsiella pneumoniae* species complex
QC: quality controlled
SCAI: Simmons citrate agar with inositol
ST: sequence type
WGS: whole-genome sequencing
WMS: whole metagenomic sequencing
