# Supplementary material for: Sequence type and strain-level detection of Klebsiella pneumoniae in culture-enriched bacterial metagenomes: comparative performance of mSWEEP and StrainGE bioinformatic tools
Source: Microb Genom. 2026 Feb 11;12(2):001638. doi: 10.1099/mgen.0.001638 (PMC12893620; doi:10.1099/mgen.0.001638)
Supplement: Uncited Supplementary Material 1. [file mgen-12-01638-s001.pdf]

Supplementary Figures

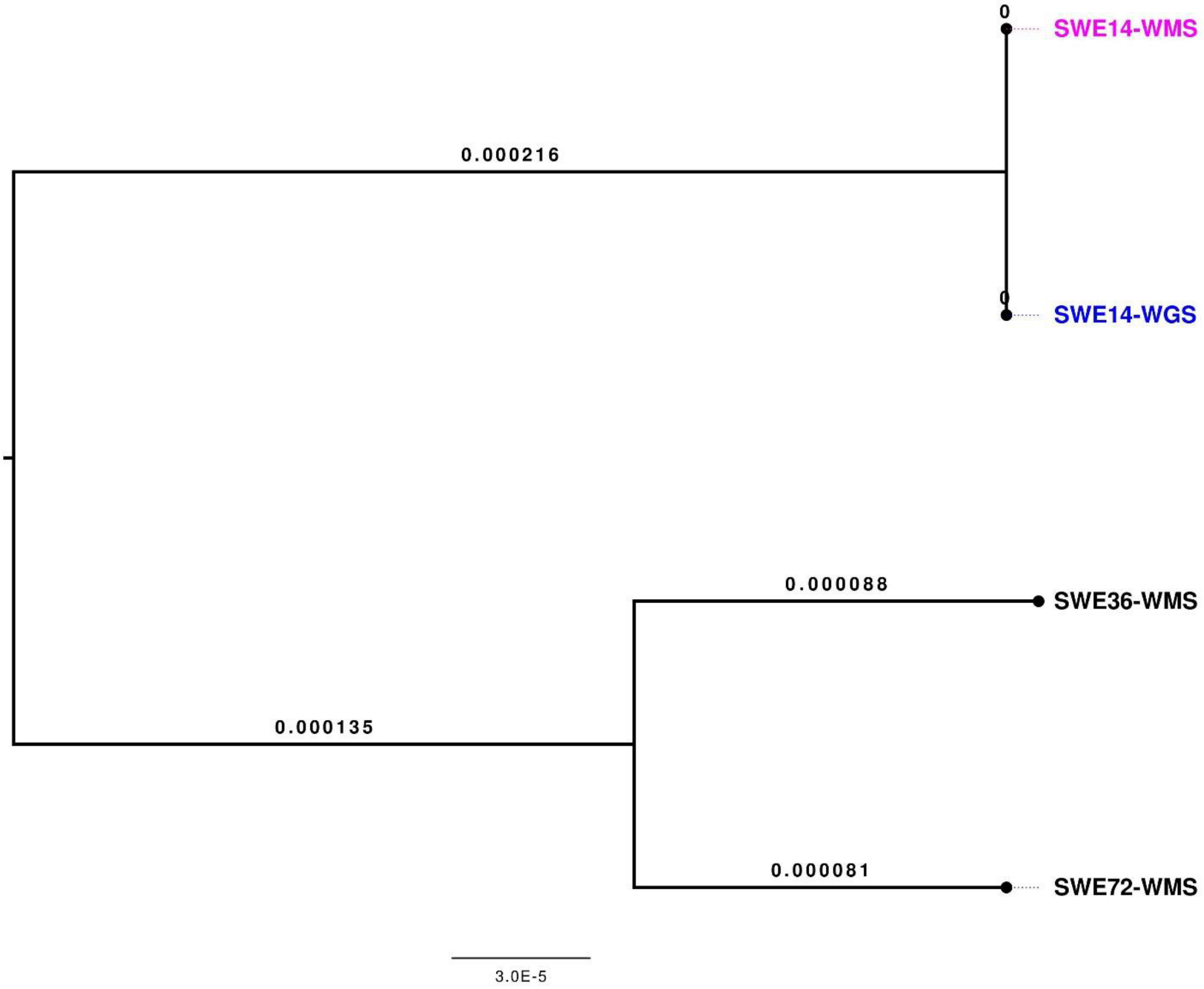

Supplementary Figure 1. Phylogenetic relationships of sweep metagenomes containing ST20 analysed by StrainGR compared to single isolate WGS SWE14 ST20. The tree was built from pairwise distances between strains based on genomic distances calculated from SNP rates via the Jukes-Cantor model.

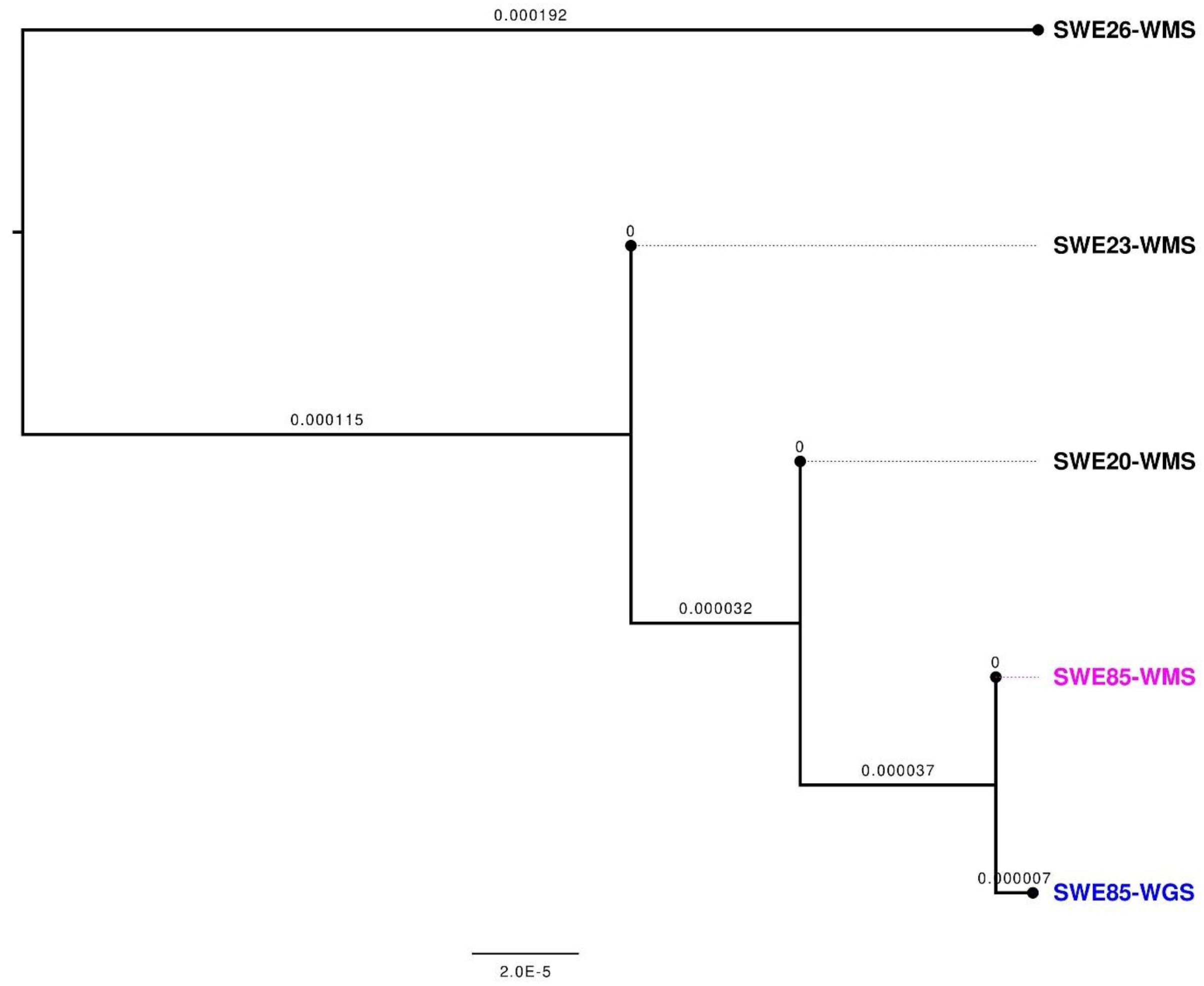

Supplementary Figure 2. Phylogenetic relationships of sweep metagenomes containing ST26 analysed by StrainGR compared to single isolate WGS SWE85 ST26. The tree was built from pairwise distances between strains based on genomic distances calculated from SNP rates via the Jukes-Cantor model

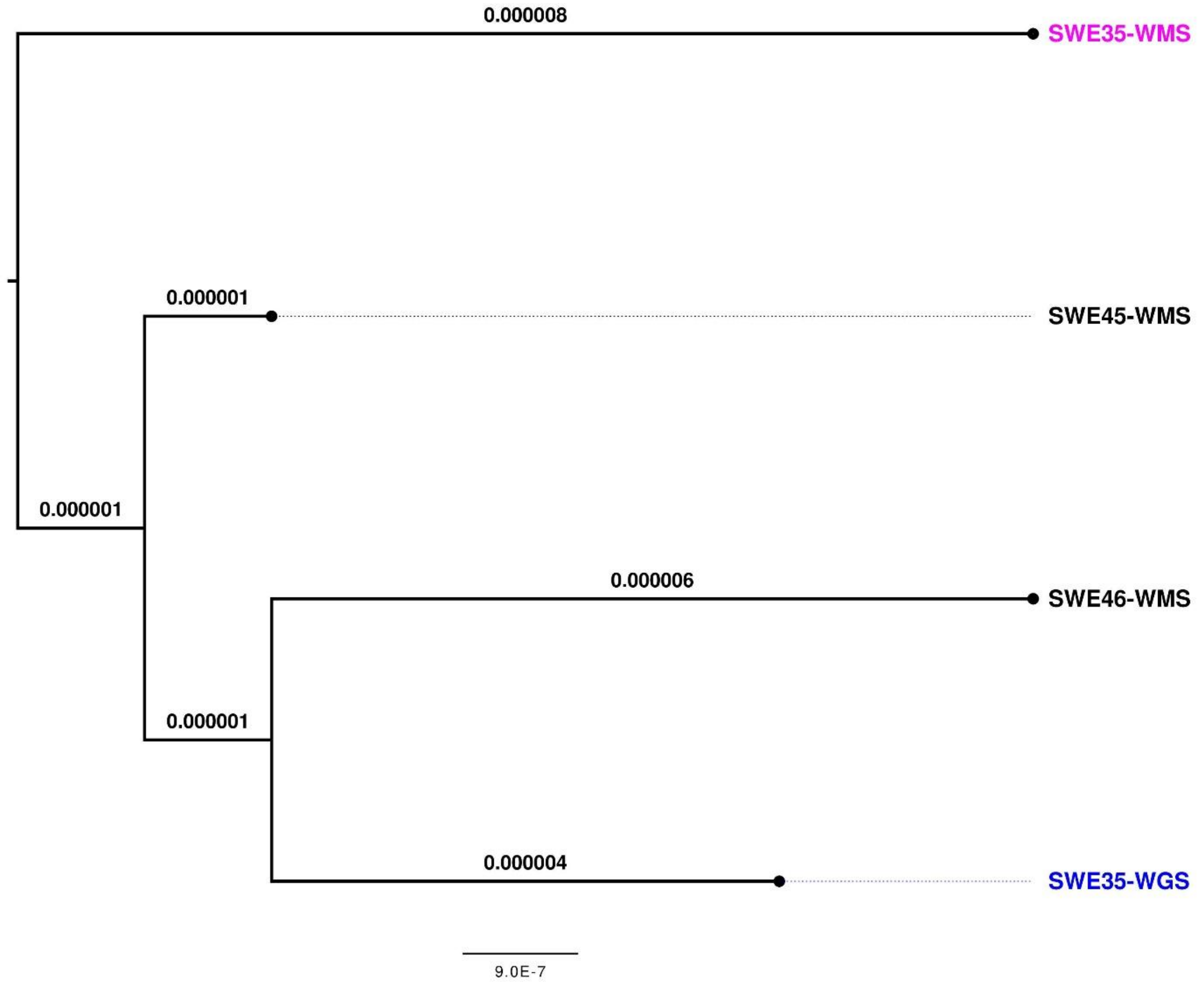

Supplementary Figure 3. Phylogenetic relationships of sweep metagenomes containing ST25 analysed by StrainGR compared to single isolate WGS SWE35 ST25. The tree was built from pairwise distances between strains based on genomic distances calculated from SNP rates via the Jukes-Cantor model

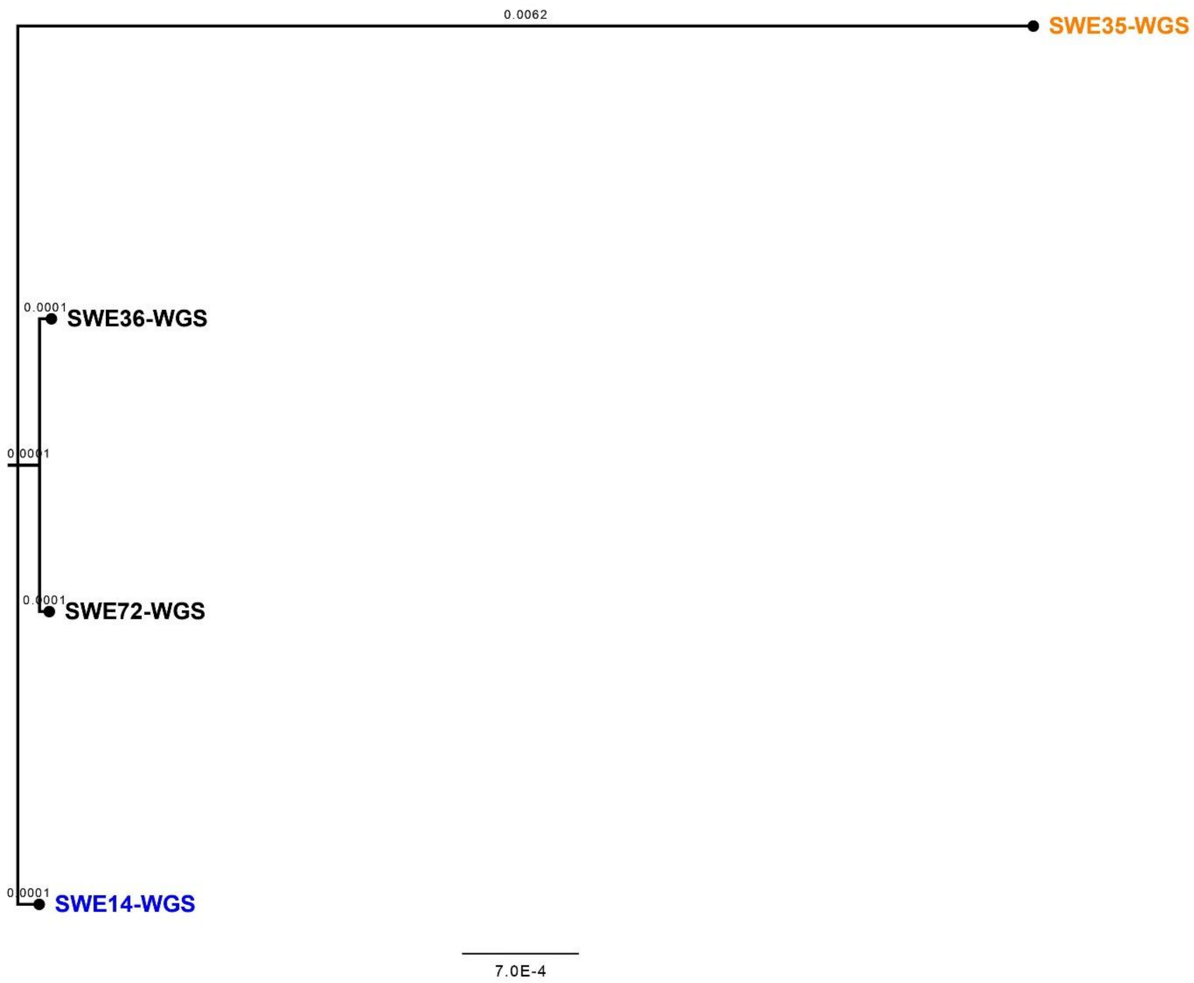

Supplementary Figure 4. Maximum likelihood (ML) phylogeny of the core genome alignment of ST20 WGS single isolates corresponding to sweep metagenomes with ST20 detected by StrainGST.

Supplementary Table 1. Comparison between T7 reference genome and mSWEEP assembled bins

Parameter	Number of Mismatch[a]	Sweep ID (Mismatch)	Number of Undetected/Unknown[b]	Sweep ID (Undetected/Unknown)
**Species**	2	8, 90	3	11, 71, 93
**STs**	0	-	4	11, 71, 90, 93
**Virulence Score**	9	34, 47, 72, 92, 94, 100, 101, 102, 103	-	-
**Resistance Score**	0	-	-	-
**Yersiniabactin**	0	-	11	13, 21, 35, 44, 45, 46, 47, 70, 72, 92, 94
**Colibactin**	0	-	3	101, 102, 103
**Aerobactin**	0	-	1	100
**Salmochelin**	0	-	1	100
**RmpADC**	0	-	-	-
**wzi**	1	97	-	-
**K_locus**	2	42, 95	-	-
**O_locus**	3	40, 75, 99	-	-
**AGly_acquired**	0	-	4	35, 40, 45, 47
**Flq_acquired**	0	-	-	-
**Sul_acquired**	0	-	5	35, 40, 43, 45, 46
**Tet_acquired**	0	-	4	35, 40, 45, 46
**Tmt_acquired**	0	-	2	35, 40
**Bla_acquired**	0	-	5	35, 36, 40, 45, 46
**Bla_chr**	1	35	3	47, 84, 88
**SHV_mutations**	0	-	2	47, 88

[a]mismatch is considered when feature is detected but differed between same STs
[b]undetected/unknown is recorded when the feature is missing either in the reference genome or in the assembled genomes

Supplementary Table 2a. Pairwise estimated genomic distances (Juke-Cantor model) between sweep samples containing ST20 with SWE14 WGS isolate as reference

	SWE14-isolate	SWE36-WMS	SWE72-WMS	SWE14-WMS
**SWE14-isolate**	0	0.00044	0.00044	0
**SWE36-WMS**	0.00044	0	0.00017	0.00044
**SWE72-WMS**	0.00044	0.00017	0	0.00043
**SWE14-WMS**	0	0.00044	0.00043	0

Supplementary Table 2b. Pairwise SNP distances among isolates belonging to ST20 corresponding to ST20 sweep samples

	SWE14 isolate	SWE36 isolate	SWE72 isolate	SWE35 isolate[a]
**SWE14 isolate**	0	1678	1432	29866
**SWE36 isolate**	1678	0	605	30136
**SWE72 isolate**	1432	605	0	30065
**SWE35 isolate[a]**	29866	30136	30065	0

[a]ST25 isolate included as an outlier

Supplementary Table 2c. Pairwise estimated genomic distances (Juke-Cantor model) between sweep samples containing ST26 with SWE85 WGS isolate as reference

	SWE85-isolate	SWE20-WMS	SWE23-WMS	SWE26-WMS	SWE85-WMS
**SWE85-isolate**	0	0.00005	0.00004	0.00043	6.8E-06
**SWE20-WMS**	0.00005	0	0.00003	0.00033	0.00004
**SWE23-WMS**	0.00004	0.00003	0	0.00031	0.00003
**SWE26-WMS**	0.00043	0.00033	0.00031	0	0.00042
**SWE85-WMS**	6.8E-06	0.00004	0.00003	0.00042	0

Supplementary Table 2d. Pairwise estimated genomic distances (Juke-Cantor model) between sweep samples containing ST25 with SWE35 WGS isolate as reference

	SWE35-isolate	SWE46-WMS	SWE45-WMS	SWE35-WMS
**SWE35-isolate**	0	0.00001	0.00001	1.32137E-05
**SWE46-WMS**	0.00001	0	0.00001	0.00002
**SWE45-WMS**	0.00001	0.00001	0	0.00001
**SWE35-WMS**	1.32137E-05	0.00002	0.00001	0

Links to Analysis Tools and Code Repositories:

1. mSWEEP: https://github.com/PROBIC/mSWEEP
2. mGEMS: https://github.com/PROBIC/mGEMS
3. PopPUNK: https://poppunk-docs.bacpop.org/
4. StrainGE: https://github.com/broadinstitute/StrainGE
5. StrainGE user manual: https://strainge.readthedocs.io/en/latest/
6. Kleborate: https://github.com/klebgenomics/Kleborate
7. Kaptive: https://github.com/klebgenomics/Kaptive
8. Kraken: https://github.com/jenniferlu717/KrakenTools
9. Kraken2: https://github.com/DerrickWood/kraken2
10. Bracken: https://ccb.jhu.edu/software/bracken/
11. snp-dists (often written as SNPdist): https://github.com/tseemann/snp-dists
12. Snippy: https://github.com/tseemann/snippy
13. RAxML (standard version): Standard: https://github.com/stamatak/standard-RAxML
14. FigTree: https://tree.bio.ed.ac.uk/software/figtree/
